# The molecular phenotype of kisspeptin neurons in the medial amygdala of female mice

**DOI:** 10.3389/fendo.2023.1093592

**Published:** 2023-02-10

**Authors:** Katherine M. Hatcher, Leah Costanza, Alexander S. Kauffman, Shannon B. Z. Stephens

**Affiliations:** ^1^ Department of Neuroscience and Experimental Therapeutics, Albany Medical College, Albany, NY, United States; ^2^ Department of OBGYN and Reproductive Sciences, University of California, San Diego, La Jolla, CA, United States

**Keywords:** kisspeptin, KISS1, GnRH, amygdala, estrogen, Cck, RiboTag, RNAseq

## Abstract

Reproduction is regulated through the hypothalamic-pituitary-gonadal (HPG) axis, largely *via* the action of kisspeptin neurons in the hypothalamus. Importantly, *Kiss1* neurons have been identified in other brain regions, including the medial amygdala (MeA). Though the MeA is implicated in regulating aspects of both reproductive physiology and behavior, as well as non-reproductive processes, the functional roles of MeA *Kiss1* neurons are largely unknown. Additionally, besides their stimulation by estrogen, little is known about how MeA *Kiss1* neurons are regulated. Using a RiboTag mouse model in conjunction with RNA-seq, we examined the molecular profile of MeA *Kiss1* neurons to identify transcripts that are co-expressed in MeA *Kiss1* neurons of female mice and whether these transcripts are modulated by estradiol (E_2_) treatment. RNA-seq identified >13,800 gene transcripts co-expressed in female MeA *Kiss1* neurons, including genes for neuropeptides and receptors implicated in reproduction, metabolism, and other neuroendocrine functions. Of the >13,800 genes co-expressed in MeA *Kiss1* neurons, only 45 genes demonstrated significantly different expression levels due to E_2_ treatment. Gene transcripts such as *Kiss1*, *Gal*, and *Oxtr* increased in response to E_2_ treatment, while fewer transcripts, such as *Esr1* and *Cyp26b1*, were downregulated by E_2_. Dual RNAscope and immunohistochemistry was performed to validate co-expression of MeA *Kiss1* with *Cck* and *Cartpt*. These results are the first to establish a profile of genes actively expressed by MeA *Kiss1* neurons, including a subset of genes regulated by E_2_, which provides a useful foundation for future investigations into the regulation and function of MeA *Kiss1* neurons.

## 1 Introduction

Reproduction is regulated by gonadotropin-releasing hormone (GnRH) secreted from neurons in the hypothalamic forebrain. Activation of GnRH neurons, and hence the reproductive axis, requires kisspeptin, a neuropeptide encoded by the *Kiss1* gene. Humans and mice with gene mutations in *Kiss1* or its receptor, *Kiss1r*, exhibit significantly disrupted reproduction, including incomplete sexual maturation and infertility (1–3). Kisspeptin treatment potently stimulates LH and FSH release (4–7) *via* direct stimulation of GnRH neurons, which express *Kiss1r* (4–11). Thus, kisspeptin is both necessary and sufficient for GnRH release and regulation of the reproductive axis. Neurons expressing *Kiss1* have been detected in several distinct brain areas, including the anteroventral periventricular (AVPV) and periventricular (PeN) nuclei (referred to as AVPV here; also commonly called the RP3V) and arcuate (ARC) nuclei of the hypothalamus, as well as in several extra-hypothalamic areas, including the medial amygdala (MeA), bed nucleus of the stria terminalis, and the lateral septum (7, 8, 12–20).

Hypothalamic *Kiss1* neurons in the AVPV and ARC have been well-studied and are known to be regulated by testosterone (T) and estradiol (E_2_) (13–15). In the ARC, sex steroids inhibit ARC *Kiss1* expression, whereas the removal of sex steroids increases ARC *Kiss1* levels, suggesting that kisspeptin neurons in the ARC are involved in sex steroid negative feedback (13–15). In contrast, in the AVPV, *Kiss1* levels are increased by sex steroids, particularly E_2_ (13–15), suggesting that AVPV *Kiss1* neurons participate in the E_2-_ mediated positive feedback that triggers the preovulatory LH surge in females (21, 22). E_2_ regulation of AVPV and ARC *Kiss1* levels occurs specifically *via* estrogen receptor α (ERα), which is highly expressed in both of these *Kiss1* populations (13, 14, 18, 23, 24). Recent RNA-seq studies of AVPV and ARC *Kiss1* neurons reported other co-expressed genes in these specific cell populations that also respond to E_2_ treatment (25–27), including hormone receptors such as *Pgr*, *Ghsr*, and *Npr2* (26, 27).

MeA *Kiss1* expression is also regulated by sex steroids. Similar to AVPV *Kiss1* expression, MeA *Kiss1* levels dramatically increase with T or E_2_ exposure in both sexes and fall to nearly undetectable levels in the absence of sex steroids (17, 18, 20, 28). However, the non-aromatizable androgen, DHT, has no stimulatory effect on MeA *Kiss1* expression (17), which suggests that the observed upregulation of MeA *Kiss1* expression by T is mediated by E_2_ signaling after aromatization; this possibility is supported by the presence of high aromatase expression in the MeA region (17, 29–31). As with *Kiss1* in the AVPV and ARC (14, 23), data from ErαKO and ERβKO mice show that the ERα receptor subtype is required for E_2_’s upregulation of MeA *Kiss1* levels (18). Despite these findings demonstrating that *Kiss1* expression in the MeA is potently stimulated by E_2_, the functional significance of this E_2_ stimulation is still currently unknown. In contrast to their E_2_-induced upregulation, MeA *Kiss1* levels are inhibited by GABA signaling through GABA_B_R. This is evidenced by very high *Kiss1* expression in the MeA, but not the AVPV or ARC, of GABA_B_R knockout mice of both sexes (19, 20). Whether this GABA effect is direct or indirect is still unknown, though MeA *Kiss1* neurons are reported to expressed GABA_B_R as determined with *in situ* hybridization (19).

At present, E_2_ and GABA, acting *via* ERα and GABA_B_R respectively, are the only known regulators of MeA *Kiss1* neurons. In fact, almost nothing is known about the identities of other receptors, co-transmitters and signaling factors, and transcription factors that are expressed by this specific kisspeptin neuron population, or whether other genes in MeA kisspeptin neurons are also altered by E_2_ signaling. This lack of knowledge of the phenotype of MeA *Kiss1* neurons has limited our understanding of possible functions of this kisspeptin population. The MeA has numerous behavioral and physiological functions, including effects on puberty, as well as reproductive physiology and behavior (32–37) and other non-reproductive behaviors (38–44). Classic studies found that lesions of the entire MeA disrupt ovarian cycles and impair E_2_ positive feedback in female rodents (35–37). Conversely, acute electrical stimulation of the MeA region of E_2-_primed ovariectomized (OVX) females induced high LH secretion (34), indicating that the MeA might facilitate E_2_ positive feedback of the LH surge. However, the mechanisms by which the MeA influences reproductive hormone release are still unknown, as is the identity of the specific MeA cell types responsible for these effects. Because kisspeptin can potently stimulate GnRH neurons (10, 11) and the MeA projects both directly and indirectly to the POA where GnRH neurons reside (45–48), it remains possible that MeA *Kiss1* neurons participate in the regulation of the reproductive axis by acting directly or indirectly on GnRH neurons. Some recent optogenetic and chemogenetic mouse studies have begun to address this possibility (39, 49–51), but this is still currently not well understood. Thus, at present, the various functional roles of MeA kisspeptin neurons remain unknown, in part due to our limited understanding of the detailed phenotype of those specific kisspeptin neurons.

Recent studies have begun to detail the molecular phenotype of the two kisspeptin populations in the hypothalamus. Using the RiboTag technique coupled with RNA-seq, we recently reported the identification of >13,000 genes that are expressed in AVPV *Kiss1* neurons of female mice (25). In that study, we also identified numerous AVPV *Kiss1* neuron transcripts that are differentially regulated by E_2_, such as *Pgr*, *Th, Cartpt*, and *Gal*. Two subsequent reports from Hrabovszky and colleagues used a different approach to examine E_2_ regulation of RNA transcripts in ARC and AVPV kisspeptin neurons (26, 27). These various RNA profiling studies provide insight into potential mechanisms of regulation of hypothalamic *Kiss1* neurons and, hence, reproductive status. However, similar RNA-seq analyses of kisspeptin neurons in the MeA have not yet been reported. In the current study, we used the RiboTag technique (52, 53), which allows for the isolation of mRNAs that are actively being translated into proteins, along with RNA-seq to identify the molecular phenotype of MeA *Kiss1* neurons of female mice under different E_2_ conditions.

## 2 Method

### 2.1 Animals

We used the RiboTag mouse model (54, 55) crossed with Kiss1Cre mice to identify actively-translated gene transcripts that are co-expressed specifically in MeA *Kiss1* neurons, following methods previously described (25, 54, 55). Briefly, RiboTag (*Rpl22^HA+^*) mice have a wild-type C-terminal exon floxed on the *Rpl22* gene that is followed by three copies of a hemagglutinin (HA) epitope sequence inserted prior to a stop codon (54). Cell-type-specific recombination can be induced by crossing RiboTag mice with a cell type-specific Cre recombinase-expressing mouse line, which leads to Cre-mediated recombination and expression of HA tags on ribosomes only in cells expressing Cre recombinase(54, 56). Here, *Rpl22^HA+^* mice were crossed with Kiss1Cre mice (courtesy of Carol Elias) (57) to generate Kiss1Cre+/*Rpl22^HA+/+^* female mice to be used for this study. Kiss1Cre+/*Rpl22^HA+/+^* mice express the HA-tagged ribosomes only in *Kiss1-*expressing cells, permitting isolation of ribosome-associated transcripts from just *Kiss1* cells in specific brain regions, such as the MeA. In addition to adult (8-10 weeks old) Kiss1Cre+/*Rpl22^HA+/+^* females, a small cohort of adult Kiss1Cre-/*Rpl22^HA+/+^* females was used as controls. These control mice do not have HA-tagged ribosomes in *Kiss1*-expressing cells and therefore, no *Kiss1* cell-specific RNA should be isolated. Tail DNA was used to genotype mice *via* polymerase chain reaction (PCR) to confirm genotypes of Kiss1Cre+/*Rpl22^HA+/+^* and Kiss1Cre-/*Rpl22^HA+/+^* mice (henceforth referred to as “Kiss^Ribo^” or “Control” mice, respectively). Additionally, any mice with germline recombination were excluded from the study.

In a separate experiment, adult Kiss1Cre/tdTomato mice were used to validate co-expression of select genes in MeA *Kiss1* neurons using immunohistochemistry and *in situ* hybridization co-detection (IHC-ISH). Kiss1Cre/tdTomato mice (“Kiss^tdTom^”) were generated by crossing Kiss1Cre mice with tdTomato mice (Ai9 strain, JAX stock #007909) (58), permitting tdtomato fluorescent reporter to be expressed exclusively in *Kiss1*-expressing cells.

All mice were housed 2-3 per cage (Kiss^Ribo^ and Control mice) or 2-5 per cage (Kiss^tdTom^ mice) in a 12hr:12hr light:dark cycle (lights off at 18:00h), with access to food and water *ad libitum*. All animal procedures were approved by local IACUC committees at the University of California, San Diego (Kiss^Ribo^ and Control mice) or Albany Medical College (Kiss^tdTom^ mice).

### 2.2 Hormone treatment and tissue processing

MeA *Kiss1* expression is known to be stimulated by E_2_ (20). Thus, all Kiss^Ribo^ and Control mice were ovariectomized (OVX) at 8 weeks of age, under isoflurane anesthesia, and pre-treated at this time with high dose E_2_ (2 mm of 1:30 E2: cholesterol powder) *via* subcutaneous Silastic capsule for 4 days. This dose of E_2_ in mice is known to increase MeA *Kiss1* expression, as well as induce E_2_ negative feedback to suppress LH levels (13, 14, 17, 18, 20, 25). This E_2_ pre-treatment was used to drive sufficient Cre expression in MeA *Kiss1* cells in all Kiss^Ribo^ mice and promote a high degree of incorporation of HA-tagged ribosomes in these neurons, regardless of subsequent E_2_ and OVX treatment conditions (25). For consistency between genotypes, Cre- controls were similarly given the E_2_ pre-treatment. All pre-treatment E_2_ implants were removed after 4 days and all mice were given 7 days to wash out any residual circulating E_2_. After the 1-week washout period, half of the mice were re-implanted with a new E_2_ Silastic capsule (E_2_ group, n = 12 Kiss^Ribo^; n=4 Controls), while the remaining females received no additional E_2_ treatment and served as the OVX group (n = 12 Kiss^Ribo^; n=4 Controls). 5 days after receiving the second E_2_ implant (or no implant), all females were euthanized between 11:00h and 14:00h. Blood was collected at this time *via* retroorbital bleed, and brains were collected fresh frozen on dry ice. Blood serum was assayed for LH concentrations to confirm low LH levels in the E_2_-treated group (indicating proper E_2_ negative feedback) and elevated LH levels in the OVX group (indicating lack of E_2_ negative feedback). Serum LH was measured *via* a highly sensitive mouse LH radioimmunoassay performed by the University of Virginia Ligand Assay Core (lower detection limit: 0.04 ng/mL; average reportable range: 0.04-75 ng/mL). As expected, E_2_-treated OVX females had significantly reduced mean LH levels compared to OVX females lacking E_2_ (0.22 ± 0.04 ng/mL vs 3.04 ± 0.22 ng/mL, respectively, p<0.05).

Fresh frozen brains were processed for RiboTag immunoprecipitation. The brain was micro-dissected on a coronal plane and 2 consecutive 400 µm thick slices spanning the MeA region were micro-punched bilaterally using a 2 mm diameter sampling tool. To ensure sufficient yield of isolated mRNA following immunoprecipitation, MeA micro-punches from n = 4 mice were pooled for each treatment (E_2_ and OVX groups). The total number of pooled samples per group were as follows: n = 3 for both OVX Kiss^Ribo^ and E_2_ Kiss^Ribo^, n = 1 for both OVX and E_2_ Cre- controls. Prior to the RiboTag immunoprecipitation, all pooled MeA micro-punch samples were stored at -80°C in 1.7 mL Eppendorf tubes.

For the ISH/IHC co-expression experiments, female Kiss^tdTom^ mice (n=3) were ovariectomized and received a similar E_2_ Silastic capsule for 5 days, as was done for the Kiss^Ribo^ mice. Brains were then collected in 4% paraformaldehyde, transferred to 30% sucrose 24 hours later, and stored at 4°C prior to slicing. Brains were then sectioned at 25 µm/slice, and sections containing the MeA mounted on SuperFrost Plus slides (Fisher Scientific), air dried, and stored at -80°C until the assay.

### 2.3 Immunoprecipitation and RNA extraction

RiboTag immunoprecipitation on pooled MeA samples from Kiss^Ribo^ and Control females was performed following published protocols (54, 56). Some modifications to the original protocol were performed to maximize isolation of ribosomes and their attached mRNA transcripts from MeA *Kiss1* neurons (25). Specifically, pooled MeA samples were homogenized in a homogenization buffer solution (72% H2O, 9.6% NP-40, 9.6% 2M KCl, 3.2% 1.5M Tris – pH 7.4, 1.2% 1M MgCl2, 2% Cyclohexamide 5mg/ml, 1% Protease Inhibitors, 1% heparin 100mg/ml, 0.5% RNAsin, and 0.1% DTT) at 3% weight by volume. The samples were then centrifuged at 10K rpm for 10 minutes at 4°C. After centrifugation, 10% of the lysate was saved in a separate tube as the “Input” sample, which contains mRNA from all cell types present in the MeA micropunch, including but not limited to *Kiss1* cells. To store the Input samples, 350µL of lysis buffer (from Qiagen Kit #74034) was added and briefly vortexed, and then the samples flash frozen and stored at -80°C. The remaining lysate was then used for the immunoprecipitation procedure. To precipitate *Kiss1* cell ribosomes and their associated RNA transcripts from each lysate sample, 0.25µL of antibody/100µL lysate of Biolegend Purified anti-Ha.11 Epitope Tag Antibody (#910501) was added to the remaining lysate volume and incubated for 2 hours on a gentle sample rotator at 4°C. Magnetic beads (Pierce Protein A/G #88803; 25µL beads/100µL sample) were washed with homogenization buffer (described above). Following the antibody incubation, the sample was transferred to the washed magnetic beads and incubated again at 4°C for 1 hour on gentle rotation. The lysate was removed by placing the sample tubes on a magnetic tube rack and the beads washed 3 times for 10 minutes each on a gentle rotator at 4°C with a high salt buffer (53.4% H2O, 30% 2M KCl, 10% NP-40, 3.3% 1.5M Tris – pH 7.4, 1.2% 1M MgCl2, 2% Cyclohexamide 5mg/ml, and 0.05% DTT), using fresh high salt buffer for each wash. Immediately following the washes, 350µL lysis buffer (Qiagen Kit #74034) was added to each sample and vortexed for 30 seconds. Sample tubes were then secured in a magnetic tube rack and the resulting lysate (termed the “IP” sample) was separated from the magnetic beads and transferred into another tube, flash frozen, and stored at -80°C. The IP samples only contain RNA from the HA-tagged ribosomes, which in this experiment were only present in *Kiss1* neurons. Thus, the IP samples from Kiss^Ribo^ mice contained RNA specific to MeA *Kiss1* neurons. To extract RNA from the Input and IP samples, the Qiagen RNeasy Plus Micro Kit (#74034) kit was used per kit instructions, and isolated RNA was stored in aliquots at -80°C until RT-PCR and RNA-sequencing.

### 2.4 Examination of *Cck* co-expression in *Kiss1* neurons using double-label *in situ* hybridization and immunohistochemistry

A double-label ISH/IHC co-detection assay was performed to examine the co-expression of *Cck*, a gene known to be expressed in the MeA (59, 60), in *Kiss1* neurons. This assay was performed on MeA-containing brain slices from adult Kiss^tdTom^ female mice (1 slice/mouse, n = 3 mice total). The assay measured co-expression of *Cck* mRNA (cholecystokinin) in MeA *Kiss1* neurons (labeled with tdtomato). To perform the co-expression assay, we used Advanced Cell Diagnostics’ RNAscope^®^ multiplex fluorescent V2 ISH assay with IHC co-detection, with the following RNAscope^®^ catalog probe: Mm-Cck-C3 – *Mus musculus* cholecystokinin (*Cck*) mRNA. For IHC detection of tdtomato, Rockland rabbit anti-RFP (red fluorescent protein, #600-401-379) primary antibody was used along with Invitrogen goat anti-rabbit IgG Alexa Fluor 594 (#A-11037) secondary antibody.

### 2.5 RT-PCR confirmation of *Kiss1* cell-specific mRNAs

**Figure 1:**
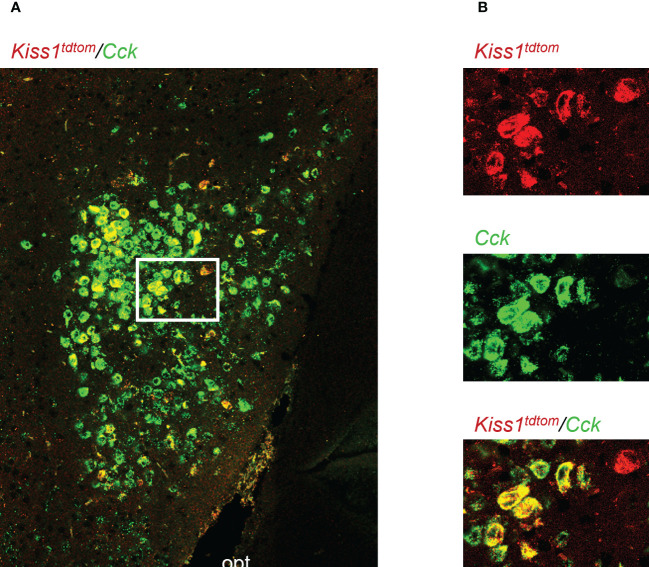
Double-label IHC/ISH of *Kiss1* ^TdTomato^ cells and *Cck* in the female mouse medial amygdala. **(A)** A representative image of *Kiss1* ^TdTomato^ cells (red) and *Cck* (green) of an E_2_-treated female mouse. **(B)** Higher magnification images of the area in the white box showing a very high degree of *Cck* mRNA (green) expression in *Kiss1* ^TdTomato^ cells (red). opt = optic tract.

To validate the efficacy of the Kiss1Cre/RiboTag method to isolate mRNA from MeA *Kiss1* neurons, we performed RT-PCR on Input and IP samples for *Kiss1* and *Cck*, which is known to be expressed in the MeA where *Kiss1* cells are located (59, 60) and was identified in the first experiment (**Figure 1**) to be co-expressed in *Kiss1* neurons. To confirm specificity, we also performed RT-PCR for *Avp*, a gene known to be expressed in the MeA, but not typically in the posterior MeA where *Kiss1* expression is observed (61). RT-PCR was performed using the iScript cDNA Synthesis Kit (Bio-Rad #1708891), per kit instructions, to synthesize cDNA from 10ng of RNA from the Input and IP samples. Using RNA-specific primers for each transcript (*Kiss1* Forward-CTGTGTCGCCACCTATGGGG; *Kiss1* Reverse- GGCCTCTACAATCCACCTGC; *Cck* Forward- CTCGGTATGTCTGTGCGTGG; *Cck* Reverse- GGTCTGGGAGTCACTGAAGG; *Avp* Forward- CCCGAGTGCCACGACGGTTT; *Avp* Reverse- CCCGGGGCTTGGCAGAATCC), the levels of *Kiss1*, *Cck*, and *Avp* mRNA in the IP and Input samples were measured *via* PCR. The PCR reaction mix included 1µL of cDNA, Jumpstart RedTaq mix (Sigma #P0982), forward and reverse primers, and RNase-free water. The PCR conditions were as follows: 94 x15’, (94 x 30”, annealing x 30”, 72 x 60”) repeat 30 times, 72 x 5’, 4 x 5’. The annealing temperature was 57°C for *Kiss1* and *Cck* and 62°C for *Avp*.

### 2.6 RNA-seq of Cre+ samples and bioinformatic analysis

RNA sequencing was performed by the Genomics Center at UC San Diego’s Institute for Genomic Medicine, using RNA from the MeA IP samples of Kiss^Ribo^ mice, for both E_2_ and OVX (no E_2_) treatments. Agilent High Sensitivity RNA ScreenTape System was used to determine the quality of all MeA IP RNA samples prior to RNA sequencing, and library preparation was only performed on IP samples with an RNA integrity number >7.6. The library was created using Illumina unstranded mRNA library kits with polyA enrichment. The Illumina HiSeq4000 platform (SR75 run type) was used to perform RNA sequencing. Raw RNA sequencing data quality control analysis was performed using Fastqc v0.11.8, then read trimming was performed using Trimmomatic 0.38, followed by alignment using STAR v2.6.0a, and then quantification of reads (RSEMv1.3.0) using GRCm38.p6/mm10 and Mus-musculus.GRCm38.98.gtf.

The Center for Computational Biology and Bioinformatics at UC San Diego performed all RNA sequencing analyses and statistics. All analyses were limited to known protein coding regions. To prepare for data exploration and preprocessing using edgeR Bioconductor package (62) written in R (63), integration and annotation of sample inputs was performed using per-gene-per-sample counts from both the count preparation and quality control were used with the per-sample RNA-seq metadata. The edgeR Bioconductor package and limma package (64) were used to explore and pre-process annotated data for determining differential gene expression specifically in transcripts produced by MeA *Kiss1* neurons. In order to eliminate low-expressing genes from comparative analyses, only transcripts with a mean CPM >1 for all 3 samples in at least one hormone treatment group (OVX or E_2_) were used for differential expression (OVX vs E_2_) and biological KEGG pathway analyses. The voom technique (65, 66), which utilizes limma and edgeR Bioconductor packages, was used to determine differential expression of any retained transcripts between the two treatment groups (E_2_ versus OVX). To test for the presence of specific genes and differential gene expression in annotated functions, pathways, and diseases, the overall data and differential gene data were analyzed with WebGestalt (67). We then performed a KEGG (Kyoto Encyclopedia of Genes and Genomes) pathway analysis (68) using PathView (69) in both E_2_ and OVX groups to examine the primary KEGG pathways observed in the overall MeA *Kiss1* RNA sequencing dataset, as well as for any pathways that were represented differently between E_2_ and OVX groups. The data set containing all identified gene transcripts is available in the Gene Expression Omnibus (geo) data repository (70).

### 2.7 Validation of gene co-expression using double-label ISH and IHC co-detection

A double-label ISH/IHC co-detection assay was performed to validate the co-expression of *Cartpt*, a gene found to be highly expressed in our RNA-seq dataset, in MeA *Kiss1* neurons. Using brain slices from 3 female Kiss^tdTom^ mice (1 slice/mouse), a double-label ISH/IHC for *Cartpt* mRNA and TdTomato, representing MeA *Kiss1* cells, was performed using Advanced Cell Diagnostics’ RNAscope^®^ multiplex fluorescent V2 ISH assay with IHC co-detection, as described previously for the detection of *Cck* in *Kiss1* cells. This assay used the RNAscope catalog probe for *Cartpt*: Mm-Cartpt-C2 – *Mus musculus* cocaine- and amphetamine-regulated transcript prepropeptide (*Cartpt*) mRNA.

The manufacturer’s protocol was followed for multiplex fluorescent V2 ISH assay with IHC co-detection for fixed frozen tissue, with some modifications to preserve tissue integrity. Briefly, slides containing brain slices with the MeA region were briefly washed with Milli-Q water, baked at 60°C for 1 hour, and post-fixed in fresh 4% paraformaldehyde for 2 hours at 4°C. The tissue was dehydrated in 50%, 70%, and 100% ethanol washes, treated with hydrogen peroxide for 10 minutes, washed with Milli-Q water, and baked for an additional 30 minutes at 60°C. Following baking, the tissue was incubated at 98-102°C for 5 minutes in 1X RNAscope^®^ target retrieval reagent, and briefly washed with Milli-Q water followed by 1X PBS-T. 200µL primary antibody (1:1000, anti-RFP) was added to each slide and slides were incubated overnight at 4°C. On Day 2, the slides were washed 3 times in 1X PBS-T, transferred to fresh 4% paraformaldehyde for 30 minutes at room temperature, followed by four washes in 1X PBS-T. 2-4 drops of Protease Plus was added to each slide and incubated at 40°C for 30 minutes, followed by 3 brief washes in Milli-Q water. Excess liquid was removed and a probe mix (Probe Diluent + RNAscope probe) was added to each slide and hybridized to the tissue at 40°C for 2 hours. The slides were washed twice with RNAscope^®^ 1X Wash Buffer at room temperature, followed by 3 amplification hybridization steps (AMP 1 and 2 for 30 minutes at 40°C, and AMP 3 for 15 minutes at 40°C) with two washes with 1X wash buffer between each amplification hybridization step. After hybridization, the HRP-C2 fluorescent signal was developed for each respective probe by adding 2-4 drops of HRP-C2 to each sample and incubating for 15min at 40°C, washing twice with 1X wash buffer, incubating with 150µL diluted Opal™ dye for 30 minutes at 40°C, and washing twice with 1X wash buffer. This was repeated again, using HRP-C3 (instead of HRP-C2) to develop the HRP-C3 fluorescent signal. The Opal™ dyes used were: Opal 520 (*Cck*) and Opal 690 (*Cartpt*). After developing the HRP-C3 signal, the 200μL secondary antibody (Goat anti-rabbit), diluted 1:100 in co-detection Antibody Diluent, was incubated on each slide for 30 minutes at room temperature, followed by two washes with PBS-T. After incubating with the secondary antibody, 4 drops of DAPI were added to each slide, incubated for 30 seconds, excess DAPI removed, and the slides then immediately coverslipped using ProLong Gold Antifade Mountant. Slides were stored at 4°C in the dark.

### 2.8 Microscopy analyses of kisspeptin and *Cck* or *Cartpt* co-expression

Using a Zeiss LSM 880 confocal microscope, images of *Cck* or *Cartpt* mRNA with TdTomato fluorescent staining were obtained at 40X (oil) magnification for one unilateral brain slice containing the MeA for each female. For each female, the number of TdTomato (reporter for kisspeptin) cells were identified (minimum 79 cells/mouse) and the number of *Kiss1* cells that co-expressed *Cck* or *Cartpt* were counted, using the RNAscope manufacturer’s criteria as a guideline (cell = ≥15 clustered dots). The mean percent of MeA *Kiss1* cells that contained *Cck* or *Carpt* was calculated.

### 2.9 Statistical analysis

T-tests were performed to compare LH levels between the OVX and E_2_-treated females. To statistically test for differences in gene expression between OVX and E_2_-treated females, the voom technique, which uses limma and edgeR Bioconductor packages (64–66), was used. This technique using simple linear regression models and produces a modified t-statistic that is interpreted like other t-statistics, with the exception that the standard errors have been moderated across genes using a simple Bayesian model (64–66). The reported *p*-value for all RNA-seq statistical analyses is an Benjamini-Hochberg-adjusted *p*-value to account for the number of genes analyzed in the dataset (71). The statistical significance was set so the adjusted *p*-value < 0.05.

## 3 Results

### 3.1 Validation and specificity of the Ribotag isolation of MeA *Kiss1* cell transcripts

Genes that are co-expressed in MeA kisspeptin neurons are unknown, though *Cck* is known to be expressed in the MeA region (59, 60), suggesting it may also be expressed specifically in kisspeptin neurons in this region. As a search for a positive control gene that is expressed in MeA kisspeptin neurons, we first performed a double-label assay to assess if *Cck* is in fact present in MeA kisspeptin neurons. Indeed, we observed that most MeA *Kiss1* cells of female mice also express high levels of *Cck* (**Figure 1**), with 88% of MeA *Kiss1* cells co-expressing *Cck* mRNA. Thus, *Cck* expression was selected as a positive control for subsequent Ribotag pulldown validation purposes.

**Figure 2:**
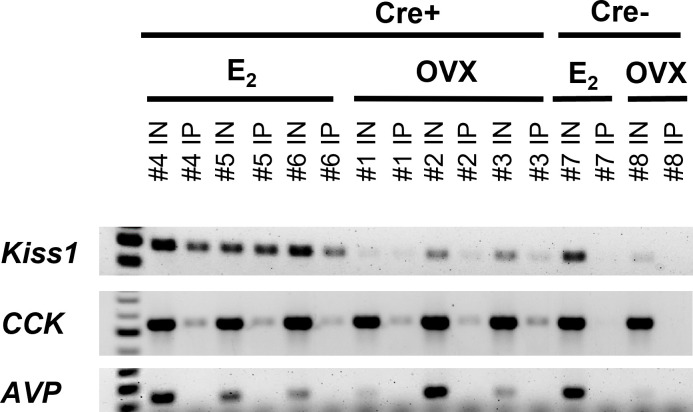
RT-PCR validation of successful isolation of ribosomal mRNA from MeA *Kiss1* cells in female mice. The number above each lane denotes the pooled sample number. Input samples contain all cells in the MeA, including, but not limited to *Kiss1* cells. IP samples contain mRNA transcripts isolated from the ribosomes of only MeA *Kiss1* cells, but not other non-*Kiss1* MeA cells. *Kiss1* and *Cck* are identified in both the Input and IP samples as expected, whereas *Avp* transcripts were located in the Input, but not the IP samples, indicating that *Avp* is not expressed in MeA *Kiss1* cells. For our Cre- control mice, the Input samples express bands for *Kiss1, Cck, and Avp*, whereas the IP samples, as expected due to the lack of the HA+ tag on the ribosomes, do not contain mRNA for these genes.

In order to validate the Ribotag isolation procedure, we performed RT-PCR using cDNA from both the Input samples (mRNA from all cells in the MeA micropunches, including *Kiss1* cells) and IP samples (mRNA from only *Kiss1* cell ribosomes) to confirm that the immunoprecipitation procedure was specific to *Kiss1* cells. As expected, both the Input and IP samples of Kiss^Ribo^ mice contained *Kiss1* transcripts (**Figure 2**). As also expected, *Kiss1* was also found in the Input samples of Control (Cre-) mice, but was not detected in the IP samples of these Controls (**Figure 2**), confirming that the immuno-pulldown was specific to *Kiss1* cells that had undergone Cre-mediated recombination to express the HA+ tag. Our positive control gene, *Cck*, was also found to be highly expressed in all the Input samples as well as in IP samples of both E_2_-treated and OVX Kiss^Ribo^ mice, but not in the IP of Control mice (**Figure 2**), further validating the selectivity of the Ribotag isolation technique. Given that little is currently known about what genes are or are not expressed in MeA *Kiss1* neurons, it is difficult to identify a true negative control. We selected *Avp* because *Avp* is expressed in the MeA region, but typically more in the rostral part of the MeA (61), whereas *Kiss1* is expressed in the more caudal (posterior) part of the MeA. Thus, AVP and *Kiss1* are in different anatomical sub-regions of the MeA and unlikely to be co-expressed in the same cells, serving as a useful negative control for our Riobotag pulldown selectivity. Indeed, here we confirmed that *Avp* mRNA was present, at low to moderate levels, in all Input samples but was absent in all IP samples (**Figure 2**), suggesting that MeA *Kiss1* cells do not express *Avp* but other MeA cell types do.

### 3.2 Identification of RNA transcripts in female MeA *Kiss1* cells

**Figure 3:**
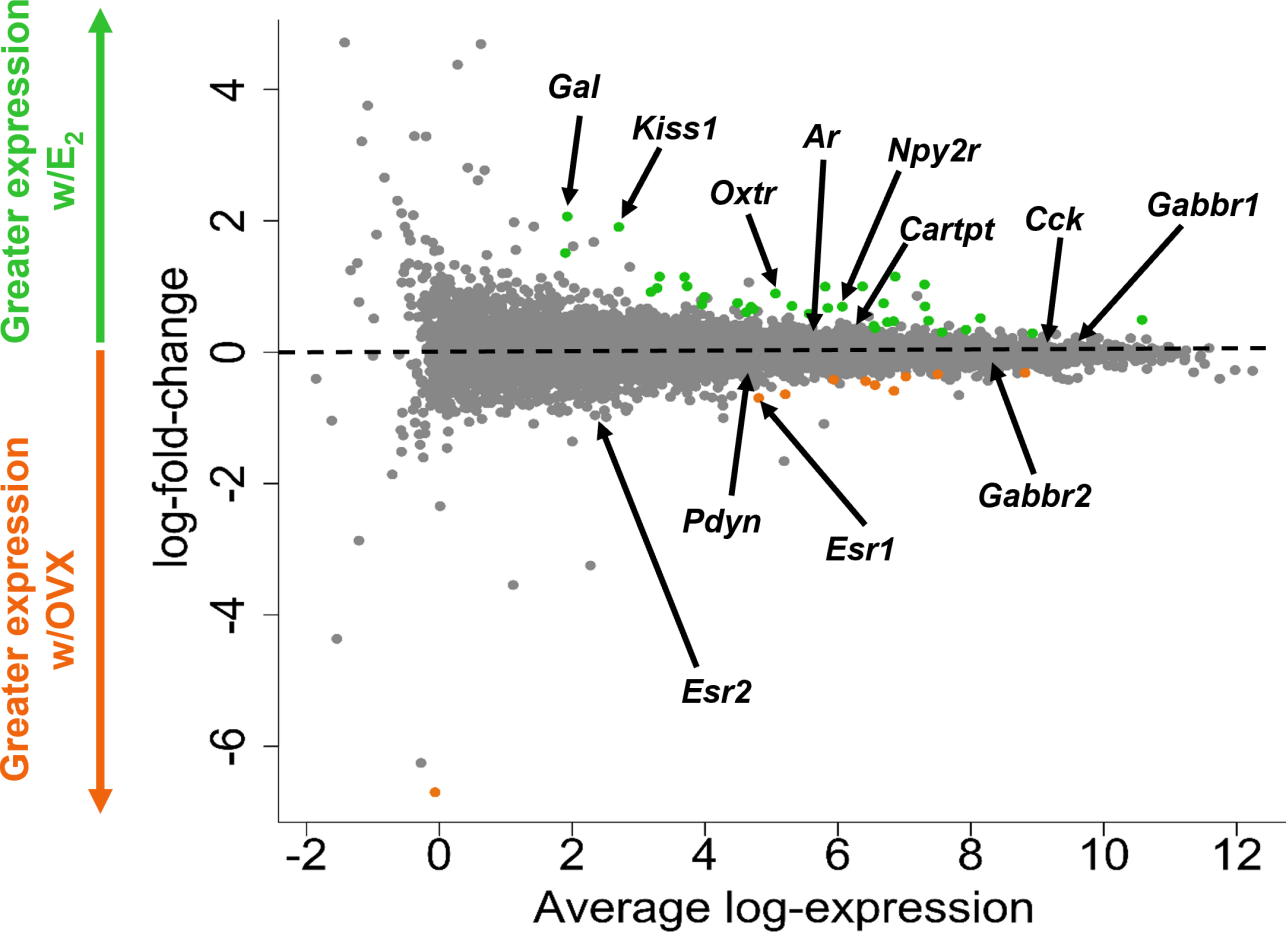
A glimma plot showing the expression of >13,800 gene transcripts identified by RNA-seq to be produced in MeA *Kiss1* cells. Each dot represents a single gene transcript. Some transcripts were upregulated by E_2_ treatment (green dots), whereas other transcripts were downregulated by E_2_ treatment (orange dots). Grey dots represent gene transcripts whose expression did not differ between OVX and E_2_ treatment groups. The y-axis represents the magnitude of difference in expression levels between OVX and E_2_ samples, with greater positive or negative values indicating a greater difference in gene expression between the two treatment groups. Y-axis values close to 0 indicate little change in expression levels between OVX and E_2_ treatments. The x-axis represents the average log-expression of each transcript, thus plotting the average of the mean expression levels of the OVX and E_2_ treatment groups. Several genes of interest are marked by arrows to indicate their location in the glimma plot.

The goal of the current study is to identify what neuropeptides, neurotransmitter synthesis and transport factors, and receptors are made by MeA *Kiss1* neurons to begin to understand the regulation and function of this *Kiss1* neuronal population. RNA quality for all E_2_ and OVX Kiss^Ribo^ samples was sufficient for RNA-seq, with RNA integrity values of at least 7.6 for all 6 Kiss^Ribo^ IP samples. As expected, the RNA quality for our Cre- control samples, which lack the HA+ tag in *Kiss1* cells, was low, <5.8. Due to the low integrity of the RNA from our control samples, only the Kiss^Ribo^ IP samples were processed for RNA-seq. The library size for each Kiss^Ribo^ sample was ≥ 25M total reads per sample, well above the 5-25M reads per sample recommended by Illumina, and well over the 10M reads aligned reach threshold, indicating a high RNA-seq quality. RNA-seq of the Kiss^Ribo^ samples identified approximately 13,800 different mRNA transcripts, including *Kiss1* and *Cck*, as well as other transcripts such as *Esr1*, *Esr2*, *Ar*, *Gal*, *Cartpt, Pdyn, Oxtr*, and *Npy2r* (**Figure 3**). Our first analysis identified the top 75 genes expressed by MeA *Kiss1* cells, regardless of E_2_ treatment. The top 3 most highly expressed genes (*Sptbn1*, *Sptan1*, and *Sptbn2*) are coding genes for spectrin proteins, which are involved in actin crosslinking, cell communication, and cell regulation. Other very highly expressed transcripts included several genes related to intra-cell signaling, such as *Gprasp1, Atp1a3, Calm1*, and *Ywhag*, genes important for protein synthesis and regulation, including *Cpe*, *Hspa8*, *Hsp90ab1*, *Eef1a1*, and *Ubb*, and genes involved with secretion and synapses, like *App*, *Syp*, and *Rtn1*. The 75 transcripts with the highest overall mean expression (*i.e.*, the mean expression of all OVX and E_2_ samples combined) in MeA *Kiss1* cells are listed in **Table 1**.

**Table 1:** The 75 genes that are most highly expressed by female MeA *Kiss1* neurons.

ENSEMBL ID	ENTREZ ID	Gene	Total Mean expression	Mean E_2_ expression	Mean OVX expression	Adjusted *p* value
ENSMUSG00000020315	20742	Sptbn1	12.242	12.099	12.384	0.5118
ENSMUSG00000057738	20740	Sptan1	11.977	11.841	12.113	0.4078
ENSMUSG00000067889	20743	Sptbn2	11.746	11.545	11.948	0.3742
ENSMUSG00000015656	15481	Hspa8	11.585	11.615	11.555	0.8581
ENSMUSG00000024617	12322	Camk2a	11.519	11.430	11.609	0.4101
ENSMUSG00000040907	232975	Atp1a3	11.462	11.438	11.486	0.8567
ENSMUSG00000001175	12313	Calm1	11.448	11.405	11.491	0.6301
ENSMUSG00000032826	109676	Ank2	11.354	11.202	11.506	0.4078
ENSMUSG00000023944	15516	Hsp90ab1	11.238	11.252	11.224	0.9463
ENSMUSG00000022892	11820	App	11.224	11.186	11.262	0.7160
ENSMUSG00000004207	19156	Psap	11.217	11.153	11.281	0.4345
ENSMUSG00000030695	11674	Aldoa	11.097	11.084	11.110	0.9524
ENSMUSG00000028833	26562	Ncdn	11.011	10.967	11.054	0.6539
ENSMUSG00000037742	13627	Eef1a1	10.981	11.022	10.940	0.6704
ENSMUSG00000019505	22187	Ubb	10.978	10.985	10.972	0.9778
ENSMUSG00000027273	20614	Snap25	10.970	11.011	10.928	0.7388
ENSMUSG00000006651	11803	Aplp1	10.909	10.885	10.933	0.8767
ENSMUSG00000031144	20977	Syp	10.902	10.905	10.899	0.9893
ENSMUSG00000036564	234593	Ndrg4	10.893	10.908	10.879	0.9240
ENSMUSG00000072235	22142	Tuba1a	10.880	10.890	10.871	0.9640
ENSMUSG00000032294	18746	Pkm	10.878	10.875	10.882	0.9902
ENSMUSG00000062825	11465	Actg1	10.875	10.878	10.871	0.9893
ENSMUSG00000014602	16560	Kif1a	10.873	10.806	10.940	0.6811
ENSMUSG00000036438	12314	Calm2	10.793	10.808	10.777	0.9524
ENSMUSG00000051391	22628	Ywhag	10.780	10.786	10.773	0.9720
ENSMUSG00000026825	13429	Dnm1	10.767	10.755	10.778	0.9527
ENSMUSG00000074657	16572	Kif5a	10.739	10.751	10.726	0.9639
ENSMUSG00000027523	14683	Gnas	10.733	10.711	10.755	0.8863
ENSMUSG00000021270	15519	Hsp90aa1	10.727	10.757	10.697	0.8383
ENSMUSG00000039953	65945	Clstn1	10.726	10.657	10.795	0.3811
ENSMUSG00000046093	170638	Hpcal4	10.702	10.662	10.742	0.7003
ENSMUSG00000019923	52696	Zwint	10.695	10.687	10.703	0.9848
ENSMUSG00000043384	67298	Gprasp1	10.681	10.689	10.674	0.9859
ENSMUSG00000027254	17754	Map1a	10.662	10.618	10.705	0.8595
ENSMUSG00000022421	73340	Nptxr	10.652	10.524	10.779	0.4542
ENSMUSG00000006930	15114	Hap1	10.642	10.740	10.544	0.3411
ENSMUSG00000021087	104001	Rtn1	10.585	10.550	10.620	0.7867
**ENSMUSG00000050711**	**20254**	**Scg2**	**10.577**	**10.823**	**10.331**	**0.0453**
ENSMUSG00000019370	12315	Calm3	10.566	10.508	10.625	0.4801
ENSMUSG00000058672	22151	Tubb2a	10.564	10.588	10.539	0.8863
ENSMUSG00000053310	64011	Nrgn	10.529	10.520	10.537	0.9784
ENSMUSG00000024758	20168	Rtn3	10.508	10.510	10.506	0.9901
ENSMUSG00000022285	22631	Ywhaz	10.507	10.501	10.514	0.9778
ENSMUSG00000015222	17756	Map2	10.500	10.539	10.461	0.8128
ENSMUSG00000004267	13807	Eno2	10.498	10.483	10.513	0.9382
ENSMUSG00000037852	12876	Cpe	10.480	10.483	10.478	0.9902
ENSMUSG00000058297	94214	Spock2	10.479	10.409	10.549	0.3330
ENSMUSG00000051853	11842	Arf3	10.439	10.397	10.481	0.6539
ENSMUSG00000025422	216439	Agap2	10.412	10.377	10.448	0.8311
ENSMUSG00000044349	319317	Snhg11	10.407	10.512	10.301	0.1504
ENSMUSG00000008348	22190	Ubc	10.393	10.406	10.380	0.9406
ENSMUSG00000026576	11931	Atp1b1	10.376	10.396	10.356	0.8799
ENSMUSG00000070802	434128	Pnmal2	10.359	10.383	10.335	0.8880
ENSMUSG00000034187	18195	Nsf	10.349	10.330	10.368	0.9145
ENSMUSG00000018707	13424	Dync1h1	10.289	10.203	10.374	0.7243
ENSMUSG00000026131	13518	Dst	10.288	10.195	10.381	0.6898
ENSMUSG00000028649	11426	Macf1	10.277	10.160	10.394	0.6331
ENSMUSG00000031517	234267	Gpm6a	10.268	10.310	10.226	0.7715
ENSMUSG00000025855	19085	Prkar1b	10.245	10.238	10.253	0.9780
ENSMUSG00000026797	20910	Stxbp1	10.237	10.210	10.265	0.8365
ENSMUSG00000052727	17755	Map1b	10.237	10.262	10.212	0.8683
ENSMUSG00000025867	12890	Cplx2	10.211	10.248	10.173	0.8091
ENSMUSG00000018965	22629	Ywhah	10.175	10.231	10.119	0.6360
ENSMUSG00000023004	22143	Tuba1b	10.171	10.225	10.116	0.6450
ENSMUSG00000059213	13199	Ddn	10.168	10.060	10.277	0.4345
ENSMUSG00000020894	22318	Vamp2	10.154	10.109	10.198	0.6710
ENSMUSG00000024121	11984	Atp6v0c	10.150	10.157	10.142	0.9713
ENSMUSG00000016349	13628	Eef1a2	10.103	10.097	10.109	0.9857
ENSMUSG00000022199	59049	Slc22a17	10.088	10.066	10.110	0.8834
ENSMUSG00000025151	94275	Maged1	10.081	10.096	10.065	0.9356
ENSMUSG00000029223	22223	Uchl1	10.073	10.068	10.079	0.9895
ENSMUSG00000025579	14387	Gaa	10.072	10.058	10.086	0.9606
ENSMUSG00000025393	11947	Atp5b	10.068	10.050	10.087	0.9085
ENSMUSG00000026223	64294	Itm2c	10.065	10.129	10.001	0.5503
ENSMUSG00000025428	11946	Atp5a1	10.064	10.058	10.070	0.9859

Transcripts are listed in descending order of total mean expression, regardless of hormone treatment. *p <*0.05 signifies significant differential expression between E_2_ and OVX conditions, denoted in bold type.

Many transcripts for genes encoding neuropeptides, receptors, and proteins involved in neurotransmitter synthesis or transport were found to be highly expressed in the RNA-seq data. In addition to *Gabbr1* and *Gabbr2* being expressed in the RNA-seq dataset, which supports previous co-expression double-label assays with GABA_B_R and *Kiss1* in the MeA (19), the RNAseq identified new transcripts that appear to be expressed by MeA *Kiss1* cells, some of which are implicated in reproductive function or other behaviors. Aside from *Kiss1*, RNAseq identified other neuropeptides implicated in modulating reproduction, metabolism, and stress, such as *Cck*, *Pdyn*, *Tac1*, *Gal*, *Cartpt*, *Trh*, *Sst*, *Npy, Agt, Vgf*, and *Penk* (**Table 2**). Transcripts related to GABA synthesis and transport, like *Vgat, Gad1*, and *Gad2*, were also expressed, as were genes important for glutamate transport and synthesis, such as *Glud1, Vglut1, and Vglut2* (**Table 2**). **Table 2** provides a more detailed list of ligand (or their related enzymes) gene transcripts identified in this RNA-seq dataset. A few ligands of interest that were absent include *Avp, C3, Calca, Nmu, Oxt, Ghrh, Gnrh1, and Tshb*.

**Table 2:** Notable ligand (or their related enzyme) transcripts identified in female MeA *Kiss1* cells.

ENSEMBL	ENTREZ ID	Gene	Overall Mean expression	Mean E_2_ expression	Mean OVX expression	Adjusted p value
ENSMUSG00000037428	381677	Vgf	9.288	9.284	9.292	0.9895
ENSMUSG00000032532	12424	Cck	9.087	9.205	8.968	0.2596
ENSMUSG00000045573	18619	Penk	8.372	8.423	8.320	0.8302
ENSMUSG00000030500	140919	Slc17a6	7.669	7.869	7.468	0.1087
ENSMUSG00000070880	14415	Gad1	7.387	7.461	7.312	0.7006
ENSMUSG00000026787	14417	Gad2	7.249	7.404	7.093	0.2441
ENSMUSG00000037771	22348	Slc32a1	6.754	6.807	6.700	0.8247
ENSMUSG00000021647	27220	Cartpt	6.442	6.510	6.374	0.9269
ENSMUSG00000004366	20604	Sst	6.222	6.176	6.269	0.7983
ENSMUSG00000005892	22044	Trh	6.136	6.025	6.246	0.7929
ENSMUSG00000024256	11516	Adcyap1	5.975	6.054	5.895	0.7793
ENSMUSG00000061762	21333	Tac1	5.436	5.505	5.367	0.8532
ENSMUSG00000021342	19109	Prl	5.189	4.431	5.946	0.8961
ENSMUSG00000031980	11606	Agt	4.788	5.110	4.466	0.3055
ENSMUSG00000027400	18610	Pdyn	4.744	4.658	4.830	0.6762
ENSMUSG00000045731	18155	Pnoc	3.968	4.173	3.763	0.5648
ENSMUSG00000029819	109648	Npy	3.635	3.674	3.596	0.9562
ENSMUSG00000024517	225642	Grp	3.578	3.564	3.593	0.9861
ENSMUSG00000019890	67405	Nts	3.024	3.114	2.935	0.8756
ENSMUSG00000019772	22353	Vip	2.973	2.823	3.124	0.7557
**ENSMUSG00000115958**	**280287**	**Kiss1**	**2.701**	**3.656**	**1.746**	**0.0049**
ENSMUSG00000037010	30878	Apln	2.643	2.689	2.597	0.9770
ENSMUSG00000020713	14599	Gh	2.274	0.780	3.767	0.8107
ENSMUSG00000025400	21334	Tac2	2.240	2.053	2.427	0.7956
ENSMUSG00000027524	13616	Edn3	2.088	2.133	2.044	0.9845
**ENSMUSG00000024907**	**14419**	**Gal**	**1.925**	**2.947**	**0.903**	**0.0207**
ENSMUSG00000000214	21823	Th	1.853	2.252	1.454	0.5648
ENSMUSG00000044988	83428	Ucn3	1.425	1.641	1.209	0.7486

Transcripts are listed in descending order based on total mean expression (average log CPM for E_2_ and GDX treatments combined). *p <*0.05 signifies significant differential expression between E_2_ and OVX conditions, denoted in bold type.

We examined transcripts for receptors in the RNA-seq data, which might provide insight into how MeA *Kiss1* neurons are regulated, either by hormones or neural signaling. Sex steroid receptors were present, as might be predicted given estrogen’s known ability to upregulate *Kiss1* in the MeA. Specifically, estrogen receptor alpha (*Esr1*), androgen receptor (*Ar*), and progesterone receptor (*Pgr*) were each moderately expressed, while estrogen receptor beta (*Esr2*) had lower expression levels (**Table 3**). Some additional receptors expressed in the RNA-seq data include *Gabbr1, Gabbr2, Cnr1, Crhr1, Crhr2, Npy1r, Npy2r*, *Npy5r, Tacr1*, and *Thra*. A more detailed list of the receptors expressed by MeA *Kiss1* cells is in **Table 3**. Of note, the following gene transcripts are for some receptors that were absent (not expressed) in the current RNA-seq dataset: *Chrna5, Ahrnb4, Pacapr1, Gpr50, Nmur2, P2rx2, Aplnr, Bdkrb2, Chrnb1, Lpar4, Npy4r, Rxfp2, Sctr*, and *Tbxa2r*.

**Table 3:** Notable receptor transcripts identified in female MeA *Kiss1* cells.

ENSEMBL	ENTREZ ID	Gene	Overall Mean expression	Mean E_2_ expression	Mean OVX expression	Adjusted p value
ENSMUSG00000058756	21833	Thra	9.906	9.842	9.969	0.4413
ENSMUSG00000024462	54393	Gabbr1	9.566	9.558	9.574	0.9644
ENSMUSG00000026959	14810	Grin1	9.173	9.137	9.209	0.7793
ENSMUSG00000033981	14800	Gria2	9.069	9.065	9.073	0.9881
ENSMUSG00000020524	14799	Gria1	8.891	8.857	8.925	0.7808
ENSMUSG00000030209	14812	Grin2b	8.877	8.803	8.951	0.6134
ENSMUSG00000039809	242425	Gabbr2	8.383	8.299	8.466	0.4427
ENSMUSG00000003378	14809	Grik5	8.311	8.299	8.324	0.9638
ENSMUSG00000001986	53623	Gria3	8.224	8.194	8.254	0.8610
ENSMUSG00000000560	14395	Gabra2	8.120	8.143	8.097	0.9145
ENSMUSG00000041380	15560	Htr2c	8.091	8.144	8.039	0.7981
ENSMUSG00000033676	14402	Gabrb3	8.053	8.051	8.056	0.9904
ENSMUSG00000010803	14394	Gabra1	7.970	8.057	7.882	0.4934
ENSMUSG00000020436	14406	Gabrg2	7.671	7.660	7.682	0.9753
ENSMUSG00000031343	14396	Gabra3	7.565	7.512	7.619	0.6177
ENSMUSG00000029778	11517	Adcyap1r1	7.560	7.550	7.571	0.9639
ENSMUSG00000032773	12669	Chrm1	7.521	7.470	7.573	0.6687
ENSMUSG00000029212	14400	Gabrb1	7.472	7.475	7.469	0.9920
ENSMUSG00000049583	108071	Grm5	7.443	7.445	7.441	0.9944
ENSMUSG00000028020	14658	Glrb	7.101	7.141	7.062	0.8005
ENSMUSG00000039579	242443	Grin3a	6.946	6.912	6.981	0.8741
ENSMUSG00000042429	11539	Adora1	6.770	6.714	6.826	0.7369
ENSMUSG00000044288	12801	Cnr1	6.736	6.694	6.778	0.7826
ENSMUSG00000059003	14811	Grin2a	6.733	6.682	6.785	0.7542
ENSMUSG00000027584	18389	Oprl1	6.618	6.664	6.572	0.8261
ENSMUSG00000027950	11444	Chrnb2	6.569	6.524	6.614	0.8002
ENSMUSG00000056755	108073	Grm7	6.550	6.572	6.528	0.9218
ENSMUSG00000030043	21336	Tacr1	6.463	6.736	6.191	0.2038
ENSMUSG00000029211	14397	Gabra4	6.410	6.465	6.355	0.7056
ENSMUSG00000020591	18217	Ntsr2	6.407	6.501	6.312	0.5123
ENSMUSG00000007653	14401	Gabrb2	6.386	6.416	6.356	0.9205
ENSMUSG00000001985	14807	Grik3	6.370	6.214	6.526	0.2038
ENSMUSG00000039059	99296	Hrh3	6.345	6.284	6.407	0.6263
ENSMUSG00000041078	14803	Grid1	6.288	6.292	6.285	0.9895
ENSMUSG00000024431	14815	Nr3c1	6.257	6.244	6.269	0.9644
ENSMUSG00000023192	108068	Grm2	6.198	6.100	6.296	0.5813
**ENSMUSG00000028004**	**18167**	**Npy2r**	**6.066**	**6.413**	**5.718**	**0.0012**
ENSMUSG00000038257	110304	Glra3	6.038	6.259	5.817	0.0828
ENSMUSG00000001260	14405	Gabrg1	5.923	6.018	5.828	0.6066
ENSMUSG00000056073	14806	Grik2	5.891	5.808	5.974	0.5503
ENSMUSG00000025892	14802	Gria4	5.847	5.921	5.773	0.6687
ENSMUSG00000045092	13609	S1pr1	5.797	5.910	5.684	0.5123
ENSMUSG00000055078	110886	Gabra5	5.759	5.751	5.766	0.9918
ENSMUSG00000021779	21834	Thrb	5.664	5.533	5.795	0.2818
ENSMUSG00000019828	14816	Grm1	5.654	5.496	5.812	0.1479
ENSMUSG00000030898	12426	Cckbr	5.648	5.632	5.664	0.9796
ENSMUSG00000046532	11835	Ar	5.591	5.771	5.411	0.0781
ENSMUSG00000044933	20607	Sstr3	5.513	5.528	5.497	0.9639
ENSMUSG00000046159	12671	Chrm3	5.489	5.423	5.554	0.7779
ENSMUSG00000033717	11551	Adra2a	5.424	5.543	5.305	0.4746
ENSMUSG00000020734	14813	Grin2c	5.393	5.379	5.407	0.9753
ENSMUSG00000029054	14403	Gabrd	5.234	5.257	5.210	0.9475
ENSMUSG00000018589	237213	Glra2	5.220	5.351	5.090	0.3503
ENSMUSG00000055026	14407	Gabrg3	5.165	5.241	5.089	0.8123
**ENSMUSG00000049112**	**18430**	**Oxtr**	**5.060**	**5.508**	**4.612**	**0.0010**
ENSMUSG00000021721	15550	Htr1a	4.978	4.945	5.011	0.9340
ENSMUSG00000031344	57249	Gabrq	4.967	4.880	5.054	0.6818
ENSMUSG00000047904	20606	Sstr2	4.965	4.803	5.128	0.2973
ENSMUSG00000045318	11553	Adra2c	4.897	4.837	4.957	0.8429
ENSMUSG00000038760	22045	Trhr	4.884	4.972	4.797	0.7172
ENSMUSG00000034997	15558	Htr2a	4.881	4.966	4.796	0.6762
**ENSMUSG00000019768**	**13982**	**Esr1**	**4.807**	**4.455**	**5.159**	**0.0264**
ENSMUSG00000035431	20605	Sstr1	4.777	4.974	4.579	0.1697
ENSMUSG00000002771	14814	Grin2d	4.718	4.798	4.638	0.7071
ENSMUSG00000071424	14804	Grid2	4.716	4.700	4.732	0.9639
**ENSMUSG00000034009**	**381489**	**Rxfp1**	**4.692**	**5.035**	**4.348**	**0.0141**
ENSMUSG00000027577	11438	Chrna4	4.679	4.699	4.660	0.9562
ENSMUSG00000031870	18667	Pgr	4.674	4.836	4.512	0.3616
ENSMUSG00000022122	13618	Ednrb	4.647	4.815	4.479	0.4312
ENSMUSG00000053004	15465	Hrh1	4.623	4.616	4.630	0.9933
ENSMUSG00000032017	110637	Grik4	4.588	4.686	4.490	0.6539
ENSMUSG00000018634	12921	Crhr1	4.511	4.454	4.568	0.8928
ENSMUSG00000021478	13488	Drd1	4.475	4.549	4.400	0.8056
ENSMUSG00000024798	15566	Htr7	4.464	4.450	4.479	0.9861
ENSMUSG00000022935	14805	Grik1	4.452	4.660	4.244	0.1504
ENSMUSG00000049511	15551	Htr1b	4.437	4.346	4.528	0.7290
ENSMUSG00000036437	18166	Npy1r	4.359	4.322	4.395	0.9433
ENSMUSG00000005268	19116	Prlr	4.320	4.529	4.111	0.6661
ENSMUSG00000035283	11554	Adrb1	4.279	4.215	4.342	0.8430
ENSMUSG00000031340	14404	Gabre	4.274	3.804	4.744	0.4340
ENSMUSG00000023964	12311	Calcr	4.269	3.855	4.683	0.2711
ENSMUSG00000003974	108069	Grm3	4.253	4.075	4.432	0.3367
ENSMUSG00000031932	14608	Gpr83	4.221	4.218	4.224	0.9961
ENSMUSG00000039106	15563	Htr5a	4.201	4.320	4.083	0.6466
ENSMUSG00000050164	207911	Mchr1	4.172	4.207	4.138	0.9424
ENSMUSG00000024211	14823	Grm8	4.111	3.976	4.246	0.6594
ENSMUSG00000020090	237362	Npffr1	3.945	3.991	3.900	0.9638
ENSMUSG00000050511	18386	Oprd1	3.848	3.765	3.931	0.8311
ENSMUSG00000025905	18387	Oprk1	3.792	3.643	3.941	0.6070
ENSMUSG00000063239	268934	Grm4	3.670	3.609	3.730	0.9026
ENSMUSG00000055737	14600	Ghr	3.668	3.835	3.502	0.6263
ENSMUSG00000027568	18216	Ntsr1	3.345	3.147	3.543	0.6070
ENSMUSG00000045613	243764	Chrm2	3.150	3.062	3.238	0.8905
ENSMUSG00000035773	114229	Kiss1r	3.141	3.158	3.124	0.9873
ENSMUSG00000050824	20609	Sstr5	3.001	3.098	2.904	0.8421
ENSMUSG00000047259	17202	Mc4r	2.493	2.699	2.287	0.6532
ENSMUSG00000021055	13983	Esr2	2.343	1.861	2.826	0.1881
ENSMUSG00000045875	11549	Adra1a	2.275	2.480	2.070	0.7479
ENSMUSG00000003476	12922	Crhr2	1.862	1.733	1.991	0.8395

Transcripts are listed in descending order based on total mean expression (average log CPM for E_2_ and GDX mice). *p <*0.05 signifies significant differential expression between E_2_ and OVX conditions, denoted in bold type.

### 3.3 E_2_-mediated differential expression of MeA *Kiss1* cell gene transcripts

**Figure 4:**
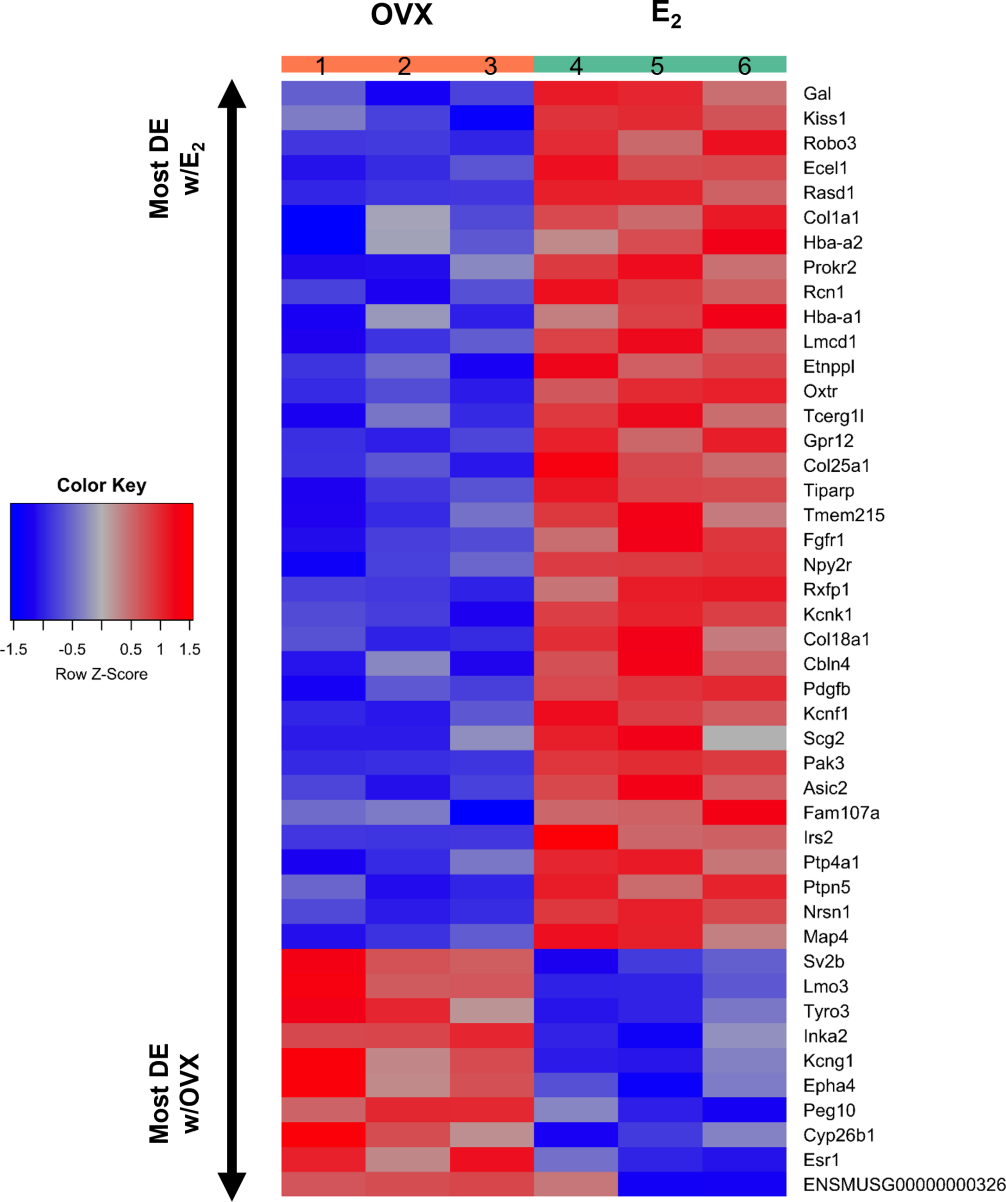
A heatmap of the 45 differentially expressed gene transcripts in MeA *Kiss1* cells. Treatment conditions and pooled sample numbers are indicated at the top. Row Z-scores demonstrate the relationship between the expression level of that transcript in the sample in comparison to the total mean expression level of that transcript. Row Z-scores that are positive (red) indicate higher expression of a transcript in a sample, in comparison to the total mean expression of the transcript, whereas Row Z-scores that are negative (blue) represent lower expression of that transcript. 35 of the gene transcripts were upregulated by E_2_ treatment, whereas only 10 of the gene transcripts had greater expression levels in OVX females.

*Kiss1* gene expression in the MeA is known to be upregulated with E_2_ treatment whereas *Kiss1* levels in the MeA are very low or absent in gonadectomized mice lacking E_2_. Thus, we hypothesized that other gene transcripts in MeA *Kiss1* cells might also change expression levels in the presence/absence of E_2_. Of the approximately 13,800 transcripts identified in the current RNA-seq data, only 45 genes had significantly different expression levels following 5-day E_2_ treatment, in comparison to OVX mice lacking E_2_ (**Table 4**; **Figure 4**). 10 of these genes were more highly expressed in OVX females (**Figure 3**, orange dots; **Figure 4**), while the remaining 35 genes were more highly expressed in E_2_-treated females (**Figure 3**, green dots; **Figure 4**). **Table 4** provides a complete list of these differentially expressed genes, while **Figure 4** is a heat map representing the expression patterns of each differentially expressed transcript for each of the 3 IP samples per treatment. As expected, *Kiss1* is more highly expressed in E_2_-treated females in comparison to OVX females (**Table 4**, **Figure 4**). In addition to *Kiss1*, E_2_ treatment upregulated transcripts encoding Galanin (*Gal*) and oxytocin receptor (*Oxtr*) as well as those for fibroblast growth factor receptor 1 (*Fgfr1*) and prokineticin receptor 2 (*Prokr2*), two genes whose loss of function results in Kallmann syndrome (72, 73), and relaxin family peptide receptor 1 (*Rxfp1*), which is implicated in regulating sperm motility, pregnancy and birth (74, 75). *Scg2* and *Ecel1* (Endothelin Converting Enzyme Like 1) are important for neuropeptide release and are also higher in E_2_-treated than OVX females. E_2_ treatment also resulted in greater expression in several genes linked to obesity and/or diabetes (insulin receptor substrate 2, *Irs2* (76); Neuropeptide Y receptor 2, *Npy2r* (77); Transcription Elongation Regulator 1 Like, *Tcerg1l* (78)), nervous system development (roundabout guidance receptor 3: *Robo3* (79)), and cancer (acid sensing ion channel 2, *Asic2* (80); Family With Sequence Similarity 107 Member A, *FAM107A* (81)). Though more gene transcripts were upregulated by E_2_, several genes were downregulated by E_2_ including *Esr1*, Cytochrome P450 Family 26 Subfamily B Member 1 (*Cyp26b1;* steroid synthesis), EPH receptor A4 (*Epha4*; nervous system development), and two genes linked to cancer (Paternally Expressed Gene 10, *Peg10*; Inka Box Actin Regulator 2, *Inka2*). Interestingly, most of the gene transcripts produced by MeA *Kiss1* cells were not found to be significantly regulated by E_2_ in the present study (**Figure 3**, grey dots; **Figure 4**).

**Table 4:** The 45 gene transcripts in MeA *Kiss1* cells that are differentially expressed due to estrogen treatment (E_2_ vs OVX).

Gene transcripts stimulated by E_2_						
ENSEMBL ID	ENTREZ ID	Gene	Total mean expression	Mean E_2_ expression	Mean OVX expression	Adjusted *p* value
ENSMUSG00000053025	64176	Sv2b	8.8167	8.6590	8.9745	0.0287
ENSMUSG00000030226	109593	Lmo3	7.5022	7.3335	7.6709	0.0300
ENSMUSG00000027298	22174	Tyro3	7.0215	6.8356	7.2074	0.0489
ENSMUSG00000092035	170676	Peg10	6.8429	6.5499	7.1360	0.0055
ENSMUSG00000026235	13838	Epha4	6.5564	6.3050	6.8078	0.0479
ENSMUSG00000074575	241794	Kcng1	6.4170	6.1943	6.6397	0.0479
ENSMUSG00000048458	109050	Inka2	5.9357	5.7267	6.1448	0.0453
ENSMUSG00000063415	232174	Cyp26b1	5.2068	4.8840	5.5295	0.0479
ENSMUSG00000019768	13982	Esr1	4.8071	4.4547	5.1595	0.0264
ENSMUSG00000117338	NA	NA	-0.0650	-3.4145	3.2846	0.0264
Gene transcripts stimulated by E_2_						
ENSEMBL ID	ENTREZ ID	Gene	Total mean expression	Mean E_2_ expression	Mean OVX expression	Adjusted *p* value
ENSMUSG00000050711	20254	Scg2	10.5768	10.8226	10.3311	0.0453
ENSMUSG00000032479	17758	Map4	8.9269	9.0713	8.7826	0.0453
ENSMUSG00000051726	382571	Kcnf1	8.1442	8.4029	7.8854	0.0012
ENSMUSG00000030854	19259	Ptpn5	7.9270	8.0982	7.7558	0.0251
ENSMUSG00000048978	22360	Nrsn1	7.5657	7.7171	7.4143	0.0287
ENSMUSG00000031284	18481	Pak3	7.3621	7.6020	7.1222	0.0010
ENSMUSG00000031565	14182	Fgfr1	7.3110	7.6590	6.9631	0.0010
ENSMUSG00000069917	110257	Hba-a2	7.3024	7.8218	6.7830	0.0441
ENSMUSG00000026247	13599	Ecel1	6.8612	7.4367	6.2858	0.0001
ENSMUSG00000020704	11418	Asic2	6.8365	7.0710	6.6021	0.0069
ENSMUSG00000021750	268709	Fam107a	6.7427	6.9725	6.5128	0.0495
ENSMUSG00000058897	77018	Col25a1	6.6862	7.0609	6.3116	0.0016
ENSMUSG00000117310	19243	Ptp4a1	6.5580	6.7396	6.3763	0.0471
ENSMUSG00000038894	384783	Irs2	6.5400	6.7415	6.3385	0.0300
ENSMUSG00000005973	19672	Rcn1	6.3696	6.8672	5.8720	0.0005
ENSMUSG00000028004	18167	Npy2r	6.0658	6.4133	5.7183	0.0012
ENSMUSG00000033998	16525	Kcnk1	5.8471	6.1833	5.5108	0.0010
ENSMUSG00000069919	15122	Hba-a1	5.8082	6.3147	5.3017	0.0158
ENSMUSG00000000489	18591	Pdgfb	5.5636	5.8579	5.2693	0.0054
ENSMUSG00000046593	320500	Tmem215	5.3082	5.6580	4.9585	0.0145
ENSMUSG00000049112	18430	Oxtr	5.0597	5.5075	4.6119	0.0010
ENSMUSG00000001435	12822	Col18a1	4.7290	5.0547	4.4034	0.0251
ENSMUSG00000034009	381489	Rxfp1	4.6916	5.0350	4.3482	0.0141
ENSMUSG00000067578	228942	Cbln4	4.6206	4.9293	4.3119	0.0479
ENSMUSG00000041468	14738	Gpr12	4.4888	4.8635	4.1142	0.0086
ENSMUSG00000091002	70571	Tcerg1l	3.9913	4.4188	3.5638	0.0264
ENSMUSG00000034640	99929	Tiparp	3.9528	4.3166	3.5890	0.0264
ENSMUSG00000050558	246313	Prokr2	3.7293	4.2241	3.2344	0.0264
ENSMUSG00000001506	12842	Col1a1	3.6909	4.2814	3.1005	0.0453
ENSMUSG00000049892	19416	Rasd1	3.3141	3.8900	2.7381	0.0075
ENSMUSG00000057604	30937	Lmcd1	3.2783	3.7636	2.7929	0.0264
ENSMUSG00000019232	71760	Etnppl	3.1841	3.6521	2.7162	0.0479
ENSMUSG00000115958	280287	Kiss1	2.7010	3.6561	1.7460	0.0049
ENSMUSG00000024907	14419	Gal	1.9249	2.9468	0.9030	0.0207
ENSMUSG00000032128	19649	Robo3	1.8956	2.6552	1.1360	0.0366

Adjusted p-value was set at 0.05. Gene transcripts are separated by those that are inhibited or stimulated by E_2_ and within each treatment, presented in descending order, beginning with the transcripts that have the highest Total Mean Expression (average expression of all E_2_ and OVX samples).

### 3.4 Biological KEGG pathways represented by gene transcripts present in MeA *Kiss1* cells

Biological pathway analysis, using KEGG pathways (68), was completed to identify potential functions of MeA *Kiss1* cells. Pathway analysis examines how sets of gene transcripts cluster together to affect various biological processes. The top biological KEGG pathways with the lowest false discovery rate (pGFdr) were identified (**Table 5**). These pathways involve signaling pathways, such as the MAPK signaling pathway, calcium signaling pathway, and neurotrophin signaling pathway. Other interesting pathways include pathways for amphetamine addiction and alcoholism, as well as those involved in regulating diseases, such as basal cell carcinoma and herpes simplex infection. There were no KEGG pathways identified that were significantly altered by E_2_ treatment.

**Table 5:** The top 10 biological KEGG pathways, regardless of estrogen status, represented by transcripts found in female MeA *Kiss1* cells.

Top 10 KEGG pathways (regardless of hormonal status)			
Pathway Name	KEGG ID	# of pathway genes expressed in MeA *Kiss1* cells	False discovery rate
Endocrine and other factor-regulated calcium reabsorption	4961	40	0.00040
Amphetamine addiction	5031	61	0.00040
Gastric acid secretion	4971	60	0.00061
Alcoholism	5034	109	0.00061
Focal adhesion	4510	185	0.00061
Glioma	5214	60	0.00061
ECM-receptor interaction	4512	72	0.00061
VEGF signaling pathway	4370	63	0.00065
Progesterone-mediated oocyte maturation	4914	75	0.00065
Prion diseases	5020	25	0.00065

The pathways shown are sorted based on the false discovery rate (pGFdr) beginning with the lowest false discovery rate.

### 3.5 Validation of RNA-seq gene expression findings using double-label ISH/IHC

**Figure 5:**
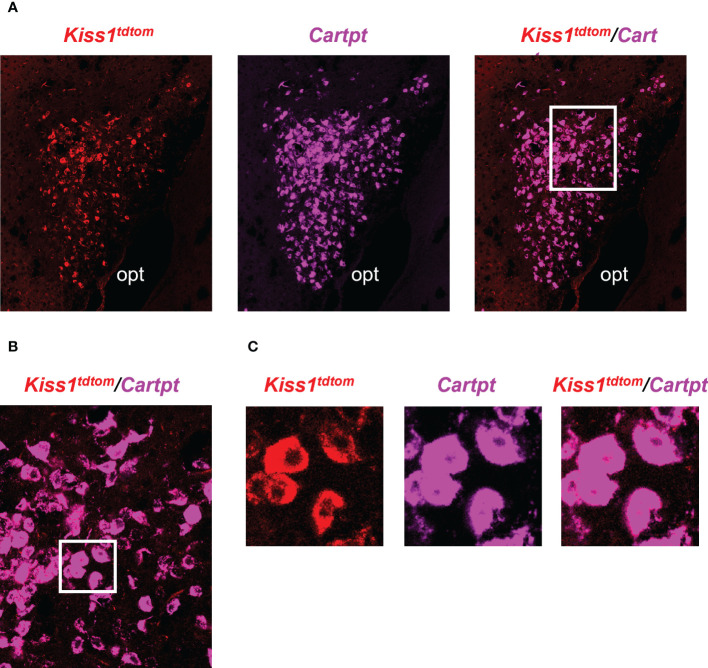
Double-label IHC/ISH of *Kiss1* ^TdTomato^ cells and *Cartpt* in the medial amygdala of an E_2_-treated female mouse. **(A)** A representative image of *Kiss1* ^TdTomato^ cells (red) and *Cartpt* (purple) of an E_2_-treated female mouse. **(B, C)** Higher magnification images of the area in the white box showing a high degree of *Cartpt* (purple) expression in *Kiss1* ^TdTomato^ cells (red). opt = optic tract.

The RNA-seq data suggested that MeA *Kiss1* cells express >13,800 gene transcripts, almost all of which have never been reported before for this specific *Kiss1* population. Therefore, in addition to validating our immuno-pulldown procedure *via* RT-PCR (**Figure 2**), we performed double-label ISH/IHC on brains from female Kiss^tdTom^ mice to confirm the co-expression of a gene identified in the RNA-seq dataset that was not previously known to be present in MeA *Kiss1* neurons (*Cartpt*; **Figure 5**, **Table 2**). We found that *Cartpt* mRNA was highly expressed in MeA brain slices, including very high overlap with MeA kisspeptin cells (tdTomato fluorescence; **Figure 5**). Quantitatively, virtually all *Kiss1* ^TdTomato^ cells (99%) in the MeA expressed *Cartpt* in this ISH/IHC assay.

## 4 Discussion

The MeA has been implicated in modulating numerous physiological and behavioral processes (32–44), including aspects of reproductive physiology. In females, lesions to the MeA disrupt ovarian cycles and prevent the E_2_-mediated LH surge, whereas acute electrical stimulation of the MeA stimulate LH release (34–37). However, the exact cellular and molecular mechanisms and specific cell types for these MeA effects on reproduction remain unknown. Kisspeptin is able to stimulate the reproductive axis, *via* direct action on GnRH neurons, but very little is known about the functions, reproductive or otherwise, of *Kiss1* neurons in the MeA, or how these neurons are regulated. Recent studies have begun to examine the detailed molecular profiles of AVPV and ARC *Kiss1* neurons, including identifying numerous gene transcripts in these populations that are regulated by E_2_ (25–27), but similar analyses have not been reported for other kisspeptin populations. In the current study, we use RNA-seq to examine the actively-translated transcripts of MeA *Kiss1* neurons to identify, for the first time, what neuropeptides, signaling factors, and receptors these neurons produce.

In this study, we used the Ribotag technique to selectively isolate actively-translated mRNAs in MeA *Kiss1* neurons. We first validated the success and specificity of this technique using RT-PCR. First, we found that both the Input (all cells in the MeA micropunch) and IP samples (only mRNAs from *Kiss1* cells) were positive for *Kiss1* mRNA from E_2_-treated females, as we would predict. Likewise, in OVX samples, *Kiss1* was detected in both Input and IP samples as expected, with lower mRNA levels (lighter bands) than for E_2_ samples given that MeA *Kiss1* expression is known to be stimulated by E_2_. The specificity of our technique was further confirmed by our samples from Cre- controls in which *Kiss1* was present in the Input samples, as expected because they contain mRNA from all MeA cells in the micropunch including *Kiss1* cells, but not in the IP samples. The lack of *Kiss1* (and *Cck*) in the Cre- IP samples indicates that our Ribotag immuno-pulldown technique was specific to isolating mRNA from just *Kiss1* cells in the micropunch. Our initial histological identification of *Cck* co-expression with *Kiss1*-TdTomato cells in the MeA (**Figure 1**) enabled us to use *Cck* as a positive control for MeA kisspeptin neurons in the RT-PCR validation step. Indeed, similar to *Kiss1*, we found that *Cck* mRNA was present in both Input and IP samples in E_2_-treated and OVX mice, with no *Cck* expression found in the IP samples of Cre- control mice, further supporting the specificity of the Ribotag technique. Knowing that *Avp* and *Kiss1* are present in different regions of the MeA, we examined our Input and IP samples for the presence/absence of *Avp* as a negative control for kisspeptin cell transcripts. Whereas Input samples contained *Avp* mRNA, indicating its presence in the MeA region, no *Avp* was detected in any IP samples as we would predict if *Avp* is not expressed in *Kiss1* cells. We also confirmed the co-expression of an RNAseq-identified gene in the Ribotag IP, *Cartpt*, in MeA kisspeptin neurons using histological assessment.

Our RNA-sequencing identified ~13,800 unique transcripts produced by MeA *Kiss1* neurons. Most of the genes with the highest expression were for general cell maintenance, neuronal signaling, or protein synthesis and regulation. These results were further supported by the data regarding biological pathway analysis (KEGG pathways), which showed that many of the gene transcripts produced by MeA *Kiss1* neurons are important for basic biological functions of neurons, such as the regulation of actin cytoskeleton, MAPK signaling pathway, and calcium signaling pathway, which are important processes for all cells, not just *Kiss1* cells. Of interest, 2 of the 5 addiction pathways, amphetamine addiction and alcoholism, were represented in the top KEGG pathways, potentially because of the heavy involvement of dopamine, dynorphin, and glutamate in these pathways. Interestingly, many of the KEGG pathways identified for MeA *Kiss1* cells were the same pathways identified for AVPV *Kiss1* cells (25), which may suggest that some of these functions may be generalized to many cells in the brain and/or suggestive of undiscovered shared functions of *Kiss1* neurons from several brain areas. We did not find any KEGG pathways that contained transcripts that were more represented in GDX or E_2_-treated mice. This result is not surprising given the low number of differentially expressed genes identified between the two hormone treatment groups, as discussed more below.

The mean expression analysis of gene transcripts in our dataset revealed >13,800 gene transcripts produced by MeA *Kiss1* neurons, of which almost all are newly identified for this specific cell population. We identified genes for several neuropeptides found in other *Kiss1* neuronal populations, such as *Vgf*, *Penk*, *Tac1*, *Pdyn*, *Pnoc*, and *Gal*, as well as several sex steroid receptors, *Ar*, *Pgr*, *Esr1*, and *Esr2*, though *Esr2* levels were much lower than the other sex steroid receptors. The actions and targets of the identified neuropeptides co-expressed in MeA *Kiss1* neurons remain unknown but may give clues into understanding the potential functions of these neurons. Interestingly, many of the identified neuropeptide genes, including the ones listed above, are also expressed in AVPV *Kiss1* neurons (25, 27), and *Kiss1* is upregulated by E_2_ in both the MeA and AVPV (13–15, 17, 18), which may indicate AVPV and MeA *Kiss1* cells share some similar functions. The presence of *Ar* in MeA *Kiss1* neurons is interesting because previous studies demonstrated that T or E_2_, but not DHT, can increase MeA *Kiss1* expression (17). However, the MeA is a sexually dimorphic brain structure, both in anatomy and genes expressed (82–84), and this is hypothesized to be produced in part by androgen signaling. Thus, it may not be surprising that MeA *Kiss1* cells (which have some sexually dimorphic qualities), express *Ar*, even if *Kiss1* expression itself is not altered by AR signaling. Interestingly, we found that *Cyp19a1*, the gene encoding for aromatase, is not expressed in MeA *Kiss1* cells, based on our current RNA-seq dataset, suggesting that the conversion of androgens to estrogens is occurring elsewhere. It is also possible that other gene transcripts in MeA *Kiss1* neurons are regulated by AR signaling, even if the *Kiss1* gene is not. Whether or not progesterone alters MeA *Kiss1* expression remains unknown, but the importance of *Pgr* in these neurons could be interesting considering *Pgr* in *Kiss1* cells is required for normal female fertility (52). Future research could evaluate whether *Pgr* specifically in MeA *Kiss1* cells influences fertility. GABA signaling through GABA_B_R is another proposed modulator of MeA *Kiss1* expression (19), and previous ISH assays demonstrated that MeA *Kiss1* neurons express GABA_B_R mRNA (19, 20). Our present RNA-seq data support this finding as transcripts for both *Gababr1* and *Gababr2* subunits were highly expressed. Importantly, the expression of receptor transcripts by MeA *Kiss1* cells does not automatically mean that the *Kiss1* gene itself is regulated *via* activation of these receptors, as the receptor signaling may influence other genes or processes in these cells.

Surprisingly, only 45 of the 13,000+ gene transcripts produced by MeA *Kiss1* neurons were found to be significantly regulated by E_2_ status, with a majority of these differentially expressed transcripts increasing with E_2_ treatment. This contrasts with AVPV *Kiss1* cells in which 683 transcripts were altered by similar E_2_ treatment (25). Regardless, it is possible that some of these E_2_-senesitive transcripts in MeA *Kiss1* neurons are involved in E_2_-regulated behavior and physiology, and future research can examine the roles of transcripts like *Gal* and *Oxtr* specifically in MeA *Kiss1* neurons in such processes. It is unknown at present if E_2_’s effects on these 45 differentially expressed genes are due to E_2_ action directly or indirectly on MeA kisspeptin cells or *via* which ER subtype the effects are mediated. Supporting the possibility of some direct E_2_ regulation, our RNA-seq dataset indicated that MeA *Kiss1* cells express *Esr1* and, to a lesser degree, *Esr2*. However, some of the differential expression of gene transcripts may be due to indirect actions of E_2_ on other afferent cells that communicate with MeA kisspeptin neurons. Because so many transcripts were not differentially expressed with E_2_ treatment, it is possible that these MeA kisspeptin neurons have primary functions that are not dependent on E_2_ status. Future research might focus on the genes that were most highly expressed in these neurons (**Tables 1**–**3**) regardless of hormone status.

Recent studies by our group and Göcz and colleagues (2022) and have begun to examine the gene transcripts made by kisspeptin neurons in the murine AVPV (25) and ARC (26), respectively, and evaluated the role of E_2_ in regulating these gene transcripts (see **Table 6** for a summary and comparison of these studies). In ARC kisspeptin neurons, 2,329 genes were found to be regulated by E_2_, with about an equal number of genes being upregulated vs downregulated by E_2_ (26). When these samples were analyzed differently with low-expressing genes removed, there were still 1,583 genes expressed by ARC kisspeptin neurons that were significantly altered by E_2_ treatment (27). In contrast to the ARC, kisspeptin neurons in the AVPV of the same mice only had 222 genes that were E_2_ regulated, with 142 of those genes being upregulated by E_2_ (27). Thus, more gene transcripts produced by ARC kisspeptin neurons appear to be responsive to E_2_ than in AVPV kisspeptin neurons. Our group previously examined gene transcripts made by female AVPV kisspeptin neurons using a different mouse model, E_2_ treatment paradigm, and RNA isolation techniques than used by Göcz and colleagues. In that prior study, we found a higher number of gene transcripts in AVPV kisspeptin neurons, 683 transcripts, that were regulated by E_2_ (25). It is possible the differences in isolation techniques (isolation from the ribosomes vs isolation from the entire kisspeptin neuron), E_2_ hormone treatment paradigms, or the parameters used to exclude low-expressing genes (summarized in **Table 6**) resulted in the different number of E_2_-regualted transcripts between these two AVPV studies. Despite these methodological differences, in both studies of AVPV kisspeptin neurons, more gene transcripts were upregulated by E_2_ than downregulated by E_2_, and over 50 of these gene transcripts that were E_2_ regulated were identified in both studies (25, 27). In the current study, we used the same mice and methodology as our prior AVPV study (25), and found that only 45 of >13,800 gene transcripts of MeA kisspeptin neurons were regulated by E_2_, with *Kiss1* being one of them. Thus, our present data suggest that, in comparison to AVPV and ARC cells, most gene transcripts produced by MeA kisspeptin neurons are not sensitive to E_2_ and may be more strongly regulated by other signaling factors. Additional research is still needed to understand how MeA kisspeptin neurons are regulated, and the present RNA-seq dataset provides a starting point for such future research.

**Table 6:** A comparison of RNA-seq studies from the MeA, AVPV, and ARC kisspeptin neuron populations.

Mouse model	MeA	AVPV		ARC		
	Current Study	Stephens et al., 2021: Endocrinology	Göcz et al., 2022: Frontiers	Göcz et al., 2022: Frontiers		Göcz et al., 2022: PNAS
	KissCre (Elias) x Ribotag mouse (HA tag on *Rpl22* gene)		KissCre (Colledge) x ZsGreen mouse			
Hormone Treatment	All mice: OVX + 4 days E_2_ Silastic capsule (pre-treatment), E_2_ capsule removal & 1 wk washout; Treatment at collection: E_2_ mice receive another E_2_ Silastic capsule (5 days), OVX mice receive no capsule.		All mice: OVX for 9 days; Treatment at collection: 4 days Silastic E_2_ capsule or OVX (sac day 13)			
Brain dissection	Micropunch of MeA or AVPV regions		laser capture microdissection of kisspeptin cells			
RNA isolation	immunoprecipitation using HA+ antibody^54^; only isolates RNA from ribosomes in *Kiss1* neurons		RNA extraction from entire kisspeptin neuron			
Samples	4 mice pooled/sample; total of 3-4 pooled samples/hormone treatment		300 kisspeptin cells/mouse, not pooled			
Library	Illumina mRNA unstranded library		TrueSeq Stranded Total RNA Library Preparation Gold kit (Illumina)			
RNA seq	Illumina HiSeq4000		Illumina NextSeq500/550 High Output kit v2.5			
Removal of low-expressing genes	CPM > 1 (comparative analysis); adjusted p-value		Basemean > 20 (comparative analysis); adjusted p-value		adjusted p-value	
Total transcripts identified	~13,800	~13,300	10623	not stated	not stated	
# total genes regulated by E^2^	45	683*	222*	1583	2329	
# genes up-regulated by E^2^	35	484	142	not stated	1190	
# genes down-regulated by E^2^	10	199	80	not stated	1139	
KEGG pathways significantly altered by E2	0	3	not stated	not stated	83	

The mouse model, methodology, analysis, and results are compared between the current study and previous work looking in transcriptomics of kisspeptin neuron populations in female mice. *indicates over 50 of the same genes regulated by E2 were found in both AVPV studies.

The current RNA-seq data greatly expands upon the limited information regarding what signaling factors and receptors are produced by MeA *Kiss1* neurons. We wanted to further validate our findings by performing an IHC/ISH for on a gene (*Cartpt*) identified for the first time by our RNAseq to be present in MeA kisspeptin neurons. This histological assay determined that virtually all of kisspeptin cells in the MeA region express *Cartpt*, supporting the very high expression levels of *Cartpt* in the RNA-seq dataset. At present, it is technically difficult to detect sufficient *Kiss1* mRNA in the MeA using RNAscope methodology owing to lower levels of *Kiss1* mRNA expressed per cell in this brain region than in the hypothalamus. One limitation in the current study is that the identification of MeA kisspeptin cells was achieved using a fluorescent reporter (TdTomato), instead of looking at actual *Kiss1* mRNA expression. When more sensitive methodology becomes available, future research should examine MeA *Kiss1* co-expression with other transcripts of interest identified in this RNA-seq dataset, to both confirm and better understand the co-expression of neuropeptides/receptors/enzymes and active *Kiss1* expression.

In summary, the current dataset is the first large-scale examination of the identities of neuropeptides, receptors, and neurotransmitter-related genes produced by MeA *Kiss1* cells. Other than being regulated by E_2_ and GABA, how *Kiss1* in the MeA is regulated remains completely unknown. The current study identified additional receptor transcripts made by these kisspeptin neurons, which could drive future research directions on the regulation of these neurons and the *Kiss1* gene itself. We also identified many neuropeptides and signaling factors not previously known to be produced by MeA *Kiss1* neurons, some of which are implicated in MeA-influenced processes like reproduction and metabolism, along with transcripts important for basic cell maintenance and survival. Interestingly, while > 13,800 transcripts are made in these cells, only 45 transcripts were significantly regulated by E_2_. Whether and how this focused E_2_ regulation is related to these neurons’ functional roles remains to be determined. Very little is known about *Kiss1* cells in the MeA and the current dataset therefore provides a valuable starting point for future research to examine the regulation and function of *Kiss1* neurons in the MeA.

## Data availability statement

The data presented in the study are deposited in the Gene Expression Omnibus data repository, accession number GSE224788.

## Ethics statement

The animal study was reviewed and approved by IACUC committees at Albany Medical College and the University of California, San Diego.

## Author contributions

The author contributions are as follows: experimental design (KMH, ASK, SBZS), data collection (KMH, LC, SBZS), data analysis (KMH, LC, ASK, SBZS), manuscript preparation and review (KMH, LC, ASK, SBZS). All authors contributed to the article and approved the submitted version.
